# Expression and Regulation of Hypoxia-Inducible Factor Signalling in Acute Lung Inflammation

**DOI:** 10.3390/cells14010029

**Published:** 2024-12-30

**Authors:** Nikolaos S. Lotsios, Chrysi Keskinidou, Sotirios P. Karagiannis, Kostas A. Papavassiliou, Athanasios G. Papavassiliou, Anastasia Kotanidou, Ioanna Dimopoulou, Stylianos E. Orfanos, Alice G. Vassiliou

**Affiliations:** 1 First Department of Critical Care Medicine, School of Medicine, National and Kapodistrian University of Athens, Evangelismos Hospital, 10676 Athens, Greece; n.lotsios96@gmail.com (N.S.L.); chrysakes29@gmail.com (C.K.); sotiriskaragiann@gmail.com (S.P.K.); akotanid@med.uoa.gr (A.K.); idimo@otenet.gr (I.D.); stylianosorfanosuoa@gmail.com (S.E.O.); 2 First University Department of Respiratory Medicine, ‘Sotiria’ Chest Hospital, School of Medicine, National and Kapodistrian University of Athens, 11527 Athens, Greece; konpapav@med.uoa.gr; 3 Department of Biological Chemistry, School of Medicine, National and Kapodistrian University of Athens, 11527 Athens, Greece; papavas@med.uoa.gr

**Keywords:** ARDS, HIF, hypoxia, acute lung injury

## Abstract

Hypoxia-inducible factors (HIFs) are central regulators of gene expression in response to oxygen deprivation, a common feature in critical illnesses. The significant burden that critical illnesses place on global healthcare systems highlights the need for a deeper understanding of underlying mechanisms and the development of innovative treatment strategies. Among critical illnesses, impaired lung function is frequently linked to hypoxic conditions. This review focuses on the expression and regulation of HIF signalling in experimental models of acute lung injury (ALI) and clinical studies in critically ill patients with acute respiratory distress syndrome (ARDS). We explore the potential dual role of HIF signalling in acute lung inflammation. Furthermore, its role in key biological processes and its potential prognostic significance in clinical scenarios are discussed. Finally, we explore recent pharmacological advancements targeting HIF signalling, which have emerged as promising alternatives to existing therapeutic approaches, potentially enabling more effective management strategies.

## 1. Introduction

It is undeniable that the global burden of critical illness is ever increasing, and to this day, many of the underlying mechanisms promoting cellular dysfunction during critical illness have not been fully elucidated. Among these, disrupted oxygen transportation, which leads to hypoxaemia and tissue hypoxia, are understood to have key roles in critical illness progression [1,2].

Hypoxaemia encompasses a life-threating development presenting with high frequency in intensive care units (ICUs) and is one of the key defining features of acute respiratory distress syndrome (ARDS) [3,4]. ARDS is a heterogenous life-threatening lung condition characterised by acute onset or worsening of respiratory symptoms, bilateral opacities in chest imaging, and non-cardiogenic respiratory failure. ARDS can be induced by a variety of causes from both direct pulmonary and indirect (extrapulmonary) lung injuries, and its current treatment options focus on supporting the patient. The most common direct stimuli are pneumonia and aspiration, while the most common extrapulmonary risk factors for ARDS are sepsis and traumatic lung injury [5]. Accurate diagnosis of the underlying cause of ARDS is of great importance for optimal treatment outcomes [6], while treatment efficacy can be affected by the administration mode [7].

The pathophysiology of ARDS involves widespread inflammation and damage to the alveolar–capillary barrier, leading to increased alveolar permeability and oedema formation [8]. As a result, gas exchange is disrupted, and hypoxaemia is established. According to the revised Berlin Definition for ARDS, the degree of hypoxaemia determines the severity of the syndrome. Patients with ARDS are categorised into three mutually exclusive categories, mild, moderate, and severe, based on the level of hypoxaemia, as measured by the PaO_2_/FiO_2_ ratio [9]. This persistent hypoxia triggers cellular and molecular responses aimed at adapting to the lack of oxygen via the hypoxia-inducible factors (HIFs) [10]. 

In this work, we review recent data regarding HIF involvement in acute lung inflammation. We will focus on clinical studies involving critically ill patients with ARDS and experimental models of acute lung injury (ALI), including lipopolysaccharide (LPS)-induced lung injury, caecal ligation and puncture (CLP)-induced lung injury, gas aspiration, ischemia/reperfusion (I/R)-induced lung injury, and ventilator-induced lung injury (VILI). Finally, we will present novel therapeutic approaches based on HIF signalling.

### Structure and Regulation of Hypoxia-Inducible Factors (HIFs)

HIFs are heterodimeric transcription factors of two uniquely regulated subunits, α and β, with significant roles in regulating gene expression under low oxygen conditions. In human tissues, the most prevalent isoform is HIF1-α. In addition, the HIF-2α and HIF-3α isoforms are active in specific cell types, namely vascular endothelial cells, type II pneumocytes, renal interstitial, and liver parenchymal cells [11,12]. Among the three isoforms, HIF-1α and HIF-2α retain key roles in regulating gene expression under hypoxia, while the function of HIF-3α has yet to be fully understood. Based on their structure, both α and β HIF subunits belong to the basic Helix–Loop–Helix–PER–ARNT–SIM (bHLH-PAS) superfamily. They present an N-terminal basic helix–loop–helix (bHLH) domain required for DNA binding, PER-ARNT-SIM (PAS) domains for heterodimerisation, and two transactivational domains (TADs) in their COOH-terminal site, in addition to oxygen dependent degradation (ODD) domains [13,14].

Even though both α and β HIF subunits are systemically expressed, their fates are highly dependent on oxygen levels, which play a key role in HIF regulation. The stability of alpha subunits is highly dependent on oxygen availability, in contrast to β subunits which are constitutively expressed. Low oxygen concentrations disrupt the function of HIF’s main repressors, thus allowing stabilised α subunits to translocate to the nucleus. This, in turn, results in the formation of the heterodimeric transcription factors, following their interaction with β subunits and the transcriptional co-activators CBP and p300 [15]. The active complex binds to specific DNA regions of genes regulated by HIF, known as hypoxia response elements (HREs), thus promoting the organism’s adaptation to hypoxia [16]. On the opposite side, under normoxia, HIF regulators are active. Initially, a group of three 2-oxoglutarate-dependent prolyl hydroxylase domain-containing proteins, known as PHDs, hydroxylate specific prolyl residues within the ODD of HIF-α subunits [17]. This modification allows the von Hippel–Lindau (VHL) E3 ubiquitin ligase to recognise the ODD domains, promoting the ubiquitylation and degradation of HIF-α subunits, and thus disrupting the HIF-mediated gene regulation [18,19]. Furthermore, another central enzyme in the regulation of HIFs is the 2-oxoglutarate-dependent factor inhibiting HIF (FIH). When oxygen levels are insufficient, FIH targets the CAD domain, hydroxylating a conserved asparagine residue. This modification hinders the α-subunit’s ability to interact with the transcriptional co-activators CBP and p300, blocking the formation of the active heterodimer [20,21,22,23].

Although oxygen availability is at the centre of HIF regulating mechanisms, several non-oxygen-dependent pathways are heavily involved in regulating the expression and stability of HIF subunits. In the inflamed lungs, HIF induction and stabilisation can be promoted through the accumulation of proinflammatory cytokines, namely tumour necrosis factor alpha (TNF-α) and interleukin (IL)-1β [24]. Established inflammation can in turn result in the generation of several reactive oxygen species (ROS), which can also induce HIF expression [25]. The nuclear factor-κΒ (NF-κΒ) signalling pathway, which is vital for the immune response to infection, presents a key role in HIF stabilisation, among others. Several interactions of NF-κΒ with both the α and β HIF subunits have been reported [26]. Stabilisation of HIF-1α as a result of exposure to bacteria is highly dependent on NF-κΒ [27,28]. Interestingly, during inflammation, not only is HIF expression promoted through NF-κΒ but also HIF itself retroactively downregulates NF-κΒ expression, thus creating a negative feedback loop [29,30]. 

**Figure 1:**
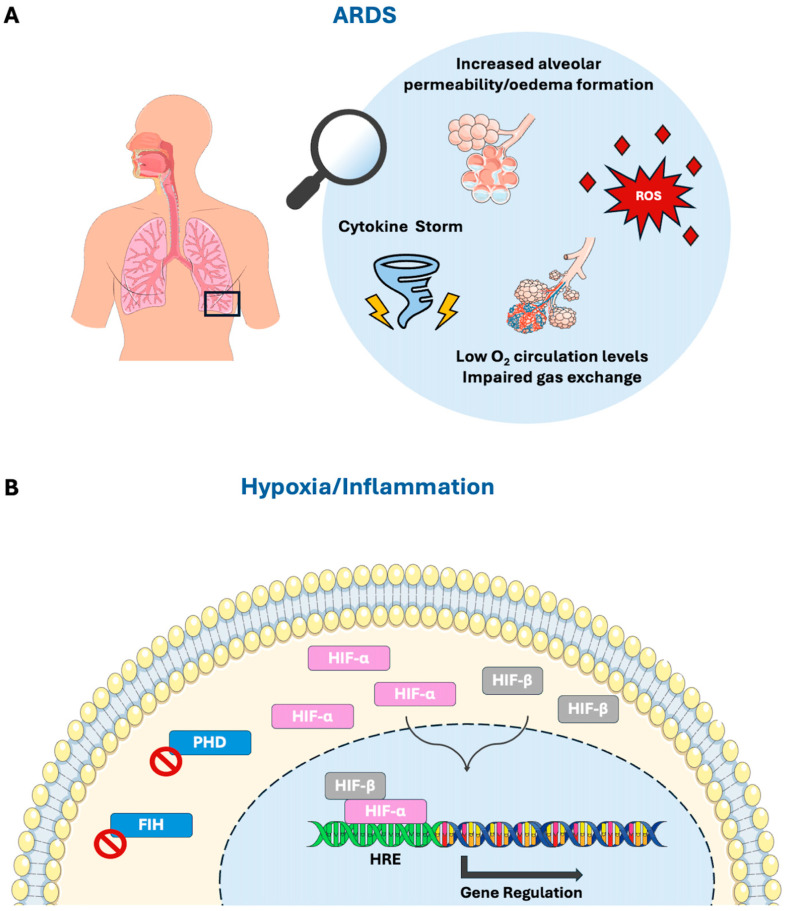
(**A**) The major pathophysiologic manifestations of ARDS. ARDS is a heterogenous and life-threatening syndrome, triggered by several causes including pneumonia, aspiration, sepsis, and trauma. This syndrome is characterised by damage to the capillary endothelium and alveolar epithelium, leading to hypoxaemia. The diffuse alveolar damage is pathophysiologically translated into a widespread inflammatory response and ROS release and a disrupted alveolar–capillary barrier, leading to increased alveolar permeability, oedema formation, and impaired gas exchange. (**B**) HIFs’ regulation pathway in hypoxic/inflammatory conditions. Low oxygen concentrations and inflammation disrupt the function of HIF’s main repressors, PHDs and FIHs, thus allowing stabilised α subunits to interact with β subunits and other transcriptional co-activators. These active complexes translocate to the nucleus and bind to specific DNA regions of genes regulated by HIF, known as HREs, thus promoting the organism’s adaptation to hypoxia. FIH, factor inhibiting HIF; HIF, hypoxia-inducible factor; HREs, hypoxia response elements; PHD, prolyl hydroxylase domain-containing proteins; ROS, reactive oxygen species.

A brief summary of the major pathophysiological manifestations of ARDS and HIF regulation in hypoxic and inflammatory conditions is depicted in Figure 1. 

HIF activation is key in several biological processes, including the regulation of cellular metabolism, inflammation, and angiogenesis. HIFs regulate genes crucial for the proper function of cellular metabolism. Specifically, under hypoxia, HIFs promote the expression of several metabolic enzymes, driving the metabolic switch from oxidative phosphorylation (OXPHOS) to glycolysis [31]. The metabolic reprogramming aims to decrease oxygen dependence, while simultaneously retaining stable ATP levels for cell survival and function. This is also achieved through increased mitochondrial degradation (mitophagy) and reduced mitochondrial biogenesis, as well as through modifications to the mitochondrial electron transport chain (ETC), which are essential in oxidative phosphorylation [32,33,34]. Moreover, genes with central roles in sepsis, known to be regulated by HIFs, exhibit a differential expression pattern in sepsis and septic shock [35]. Importantly, HIFs are tightly involved in the metabolic regulation of immune cells, enhancing their immune activity and pointing to HIFs’ central role in inflammation. Accumulation of HIFs promotes the proliferation of innate immune cells including macrophages, neutrophils, and dendritic cells, among others. Activation of macrophages under normoxic or hypoxic conditions is dependent on HIF-1α. Specifically, overexpression of HIF-1α has been shown to promote glycolytic M1-type macrophages, in contrast to HIF-2α, which promotes the anti-inflammatory M2 polarisation of macrophages [36,37]. Additionally, in the case of neutrophils, increased HIF-1α stabilisation has been strongly associated with the formation of neutrophil extracellular traps (NETs) [38,39]. Exacerbated NET formation may result in excessive inflammation; thus, the presence of a balance in neutrophil activation is important. The main mechanism in regulating neutrophil survival under hypoxia is apoptosis, and evidence supports that HIF-1α may contribute to its inhibition [40,41]. Furthermore, differentiation of dendritic cells towards a proinflammatory phenotype is driven by the stabilisation of HIF-1α, thus promoting cell migration and activation of other immune cells [42,43,44].

## 2. HIF Expression and Function in Experimental Acute Lung Injury Models

Researchers have utilised various experimental animal models of acute lung injury to explore the underlying pathophysiological processes of ALI. While no existing animal model can perfectly mimic ARDS, they provide crucial information about the underlying mechanisms of acute lung injury. Matute-Bello and colleagues describe in detail the advantages and disadvantages of each experimental ALI model and emphasise how careful selection of the ALI model is critically important, as it directly influences the accuracy and relevance of research findings [45]. Among the existing experimental models for acute lung injury, exposure of animals to endotoxins is the most applied method. Administration of LPS, a glycolipid found in the outer membrane of Gram-negative bacteria, results in a systemic response in the host, including a cytokine storm and changes in the haemodynamic equilibrium, which, however, do not reproduce the changes seen in human sepsis. Caecal ligation and puncture is widely used in mimicking sepsis-induced lung injury. Experimentally generated peritonitis promotes the formation of lung oedema due to hyperpermeability, neutrophil-mediated damage, and hypoxia, thus inducing lung injury. Furthermore, aspiration of chemical compounds with low pH or gastric fluids causes direct damage to the airway epithelium. Among the adverse effects of acid-induced lung injury are impaired fluid transportation, alveolar haemorrhage, and pulmonary oedema formation. In addition, alteration from an ischemic period to a reperfusion period results in lung injury. The I/R lung injury model is of great clinical importance, as it mimics lung complications that may arise during lung transplantation, pulmonary thromboendarterectomy, and oesophagectomy. Finally, in the VILI model, application of mechanical ventilation results in inflammation of the alveolar epithelium and activation of the capillary endothelial cells, thus disrupting the pulmonary endothelium [46]. 

### 2.1. HIF Expression in Experimental Acute Lung Injury Models

HIF expression has been investigated in several animal and cell models of acute lung injury. One of the most widely used experimental models for the induction of acute lung injury is through exposure to LPS. Both mRNA and protein expression of HIF-1α have been found to be significantly elevated in lung tissues of mice exposed to LPS [47,48,49,50]. On the contrary, serum HIF-1α levels remained unaltered in pigs exposed to LPS, while HIF-2α serum levels were decreased at 3 hours post-injury [51]. Wang and colleagues recently demonstrated that treatment of the type II alveolar epithelial cell line A549 with LPS resulted in increased HIF-1α protein expression [52]. Similarly, when isolated neutrophils from healthy humans were exposed to LPS, HIF-1α expression increased, both at the mRNA and protein levels [53].

Few studies have also explored HIF expression in CLP-induced acute lung injury. HIF-1α and HIF-2α were shown to follow different expression patterns in lung tissues of CLP-mice. Specifically, while *HIF1A* mRNA increased, the opposite was true for the endothelial PAS domain protein 1 gene (*EPAS1*), the gene encoding HIF-2α [54]. Additionally, lung tissues of CLP-mice have been shown to present increased HIF-1α protein levels [52,55].

Furthermore, lung contusion induced by thoracic trauma is a major contributing factor to the development of ARDS. Following aspiration of hydrochloride, lung lysates collected from mice presented elevated HIF-1α mRNA and protein expression [56,57]. Interestingly, the induction of HIF-1α expression is evidenced to have a key role in lung injury. In a separate set of experiments, Suresh and colleagues reconfirmed this by demonstrating reduced cytokine levels, as well as reduced accumulation of macrophages and neutrophils, in the bronchoalveolar lavage (BAL) of mice with the floxed *HIF1A* gene, following gas aspiration [57].

Expression regulation of HIF-1α has also been explored in I/R-induced acute lung injury in vivo models. Specifically, studies carried out by various groups have demonstrated significant increases in pulmonary HIF-1α protein levels in both mice and rats following I/R [58,59,60].

**Figure 2:**
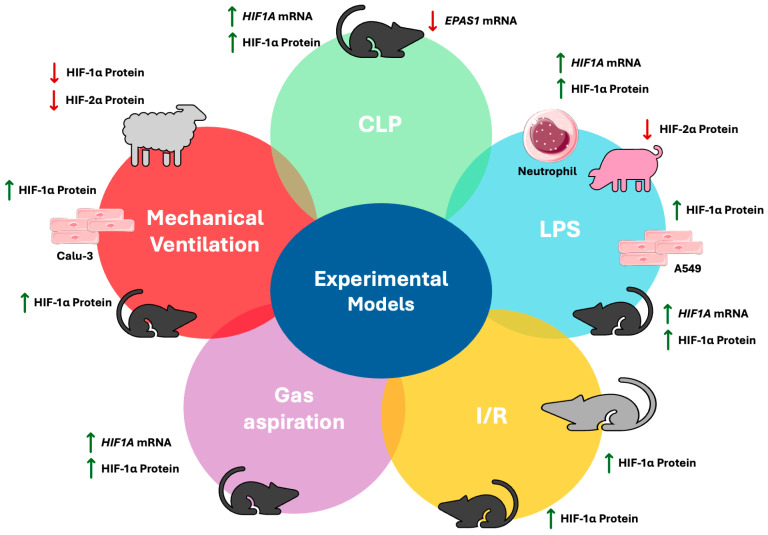
HIF-1 isoform expression in experimental models of acute lung injury. Green arrows represent expression upregulation and red arrows depict downregulation. A549, type II alveolar epithelial cell line; Calu-3, human airway epithelial cells, CLP, caecal ligation puncture; EPAS1, endothelial PAS domain protein 1; HIF, hypoxia-inducible factor; I/R, ischemia–reperfusion; LPS; lipopolysaccharide; VILI, ventilator-induced lung injury. Aspects of this figure were adapted with permission from Servier Medical Art library, available under Creative Commons license.

Of note, the expression of both HIF-1α and HIF-2α has been studied in preterm lambs that developed neonatal respiratory distress syndrome. Protein expression of both isoforms was reduced in the mechanical ventilation-induced lung injury group when compared against non-injured preterm lambs. Although the expression profile was similar to that of term gestation animals, it is evident that HIF expression serves different goals at different stages of development, with repression of HIF signalling in the preterm lung resulting in debilitating repercussions [61]. Increased HIF-1α protein has also been demonstrated in VILI mice, as well as in pulmonary airway cells following cyclic mechanical stretching [62]. An overview of experimental studies focusing on HIF-1 isoform expression in models of acute lung injury is presented in Figure 2 below.

### 2.2. HIF Regulation and Inflammation in Experimental Acute Lung Injury Models

As stated above, HIF signalling retains key roles in several biological processes. Among those, central is its implication in regulating the inflammatory response. Huang and colleagues investigated the effect of *HIF1A* silencing in CLP-mice. To this end, *HIF1A* floxed/floxed (f/f) mice were generated. Following CLP, conditional knockout mice demonstrated a persistent increase in myeloperoxidase activity in BAL, indicative of neutrophil sequestration, as well as high levels of the proinflammatory cytokines TNF-α and IL-6. Furthermore, CLP induced the expression of the forkhead box protein M1 (FoxM1), a molecule involved in vascular repair and proliferation of endothelial cells. An increase in FoxM1 expression was not evident in conditional knockout mice. Restoration of FoxM1 expression in *HIF1A* f/f mice rescued the impaired proliferation of pulmonary vascular endothelial cells, thus demonstrating the crucial role of HIF-1α in mediating vascular repair and inflammation through FoxM1 [54].

In a following study, Suresh and colleagues focused on examining the effect of HIF-1α on the inflammatory process. For this, lung injury was induced in mice through gastric aspiration, and conditional knockout mice were generated. Mice carrying floxed *HIF1A* genes presented significantly lower cytokine and chemokine levels in BAL, while *IL1B* and *IL6* gene expression, as well as mRNA expression of the NLR family pyrin domain containing 3 (*NLRP3*) inflammasome, were also reduced significantly. Moreover, *HIF1A* (+/+) mice demonstrated increased numbers of neutrophil and macrophage infiltrates in alveolar spaces. Interestingly, knockout mice presented decreased NF-κB activation in comparison to wild-type mice in both lung lysates and type II alveolar epithelial cells, thus demonstrating the interplay between HIF-1α and NF-κΒ in regulating inflammatory response following gastric aspiration [63].

Berg and colleagues aimed at exploring whether inflammatory events during LPS-induced lung injury could be orchestrated by neuronal guidance proteins. Among the targets examined, the investigators identified increased expression of netrin-1 (Ntn1) in macrophages exposed to LPS, which is dependent on HIF-1α expression. Conditional genetic deletion of *HIF1A* in myeloid cells prevented netrin-1 induction following exposure to LPS. Furthermore, *NTN1*^loxp/loxp^ mice exposed to LPS presented far more exacerbated lung injury, accompanied by higher albumin, IL-1β, and IL-6 levels in BAL as well as increased cell infiltration of white blood cells. The combined data support the role of netrin-1 in mitigating excessive pulmonary inflammation [64].

Moreover, a recent study explored the implication of SUMO-specific peptidase 3 (SENP3), a molecule known to participate in HIF-1α stabilisation in ARDS. He and colleagues exposed *SENP3*^fl/fl^ and *SENP3* conditional knockout (cKO) mice to LPS and investigated for possible links between SENP3, macrophage polarisation, and lung injury. *SENP3* cKO mice presented less alveolar thickening, lower macrophage M1 polarisation, and decreased expression of proinflammatory cytokines following LPS exposure. In summary, the investigators demonstrated the impact of SENP3 in promoting lung injury through the HIF-1α/pyruvate kinase M2 (PKM2) axis [65].

### 2.3. HIF Regulation and Metabolism in Experimental Acute Lung Injury Models

Metabolic processes during hypoxia are tightly connected to HIF signalling. Several investigators have explored the effect of HIF-regulated metabolic reprogramming on lung injury. 

In a study published in 2013 by Eckle and colleagues, the investigators sought to examine whether mechanical ventilation promotes molecular pathways crucial for lung protection. To this end, the investigators utilised both an in vitro model of cyclic mechanical stretching and an in vivo model of VILI. Both experimental models resulted in increased HIF-1α stabilisation. In Calu-3 pulmonary airway cells, inhibition of *HIF1A* expression resulted in decreased glycolysis following mechanical stretching. Interestingly, the investigators proposed that HIF-1α stabilisation as a result of stretch-induced lung injury retains a key role in repressing lung inflammation through HIF-dependent metabolic reprogramming of glucose uptake and glycolysis, thus composing an endogenous protective mechanism [62]. 

A subsequent study carried out by Tojo and colleagues expanded on the aforementioned findings in both in vitro and in vivo LPS-induced lung injury models. Murine alveolar epithelial cells and mice were treated with dimethyloxalylglycine (DMOG), a known prolyl hydroxylase (PHD) inhibitor, following LPS exposure. DMOG treatment promoted HIF-1α stabilisation, enhanced glycolysis, and attenuated ATP decline. Interestingly, this metabolic switch promoted by DMOG was highly dependent on HIF-1α expression, raising the possibility that HIF-1α-mediated glycolysis promotion could retain a vital role in protecting alveolar epithelial cells from lung injury [66]. 

Moreover, in a recent study, Woods and colleagues utilised an influenza-induced lung injury model to evaluate the metabolic pattern of tissue-resident alveolar macrophages. Exposure to hypoxia resulted in dose-dependent HIF-1α stabilisation, which in turn promoted glycolysis in alveolar macrophages. In addition, hypoxia was shown to increase glycolytic metabolites and promote changes in the cytokine production of alveolar macrophages as a response to LPS treatment. More importantly, HIF-1α stabilisation resulted in decreased death rates of alveolar macrophages following inhibition of mitochondrial respiration, indicating that HIF-1α induction could be of value in therapeutic approaches against ARDS [67].

### 2.4. HIF Regulation and Vascular Remodelling in Experimental Acute Lung Injury Models

In 2017, McClendon and colleagues proceeded to examine the role of HIF-1α in wound healing in an in vitro LPS-induced lung injury model. Rat type II alveolar cells and MLE-12 cells demonstrated increased wound closure following HIF-1α stabilisation. Furthermore, knockdown of the *HIF1A* gene in alveolar cells impacted wound healing and resulted in defective cell proliferation. Interestingly, McClendon and colleagues further attributed the increased wound healing in the alveolar cells to HIF-dependent upregulation of the chemokine receptor CXCR4 and its ligand, stromal cell-derived factor (SDF)-1 [68].

In a hypoxic pulmonary hypertension (HPH) model, a common type of pulmonary arterial hypertension that belongs to Group 3, deletion of the aquaporin 1 gene (*AQP1*), resulted in reduced proliferation, apoptosis resistance, and migration, while repressing the stability of the HIF-1α protein [69]. The authors concluded that *AQP1* deletion could, therefore, attenuate the vascular remodelling seen in HPH. This interaction between AQP1 and HIF1A was also shown in a cellular LPS-induced lung injury model, in which *HIF1A* could modulate the LPS-induced changes observed in AQP1 [70]. 

Finally, in 2022, Li and colleagues investigated the regulation of isthmin-1 (ISM1) expression, a known potent modulator of vascular permeability, under conditions of established hypoxia. Isthmin-1 had previously been shown to be upregulated in the lungs of mice exposed to LPS [71]. Expression of ISM1 was shown to be tightly related to hypoxia exposure in alveolar epithelial cells, as silencing of *HIF1A* suppressed ISM1 upregulation. Interestingly, this upregulation of ISM1 in alveolar endothelial cells was found to promote the hypoxia-induced increase in the permeability of pulmonary microvascular endothelial cell (PMVEC) monolayers, providing a greater understanding of the involvement of HIF in the regulation of hypoxia-induced pulmonary microvascular endothelial hyperpermeability [72].

Table 1 summarises the findings on HIF isoform expression in experimental models of acute lung injury.

## 3. HIF Expression in Clinical Studies in Critically Ill Patients with ARDS

As is evident above, studies performed with experimental models aim at elucidating the complex mechanisms underlying HIF function and regulation in acute lung injury. Nonetheless, clinical studies are of equal importance, providing information that, combined with data extracted from experimental models, could expose crucial aspects of HIF’s implication in the clinical setting.

In an attempt to uncover the mechanisms governing HIF regulation in ARDS, Chen and colleagues investigated the effect of the secreted phosphoprotein 1 (SPP1) on HIF-1α. Overexpression of SPP1 in blood CD4+ T cells isolated from ARDS patients decreased the expression of VHL, the main driver of HIF-1α ubiquitylation, resulting in increased HIF-1α protein expression and thus aggravating ARDS [47]. Fewer studies have focused on HIF-1α blood mRNA expression and serum levels. Recently, HIF-1α levels were evaluated in blood serum samples of ARDS patients. Results showed a significant increase in HIF-1α levels in patients compared to healthy controls. When divided into survivors and non-survivors, the latter group presented elevated HIF-1α levels compared to the former, illustrating a significant ability to predict adverse outcome in ARDS patients [73]. In 2022, Hussain and colleagues designed a case-control study demonstrating a progressive increase in HIF-1 serum levels, according to ARDS severity in COVID-19 patients [74]. HIF-1 serum levels were also significantly higher in coronavirus disease 19 (COVID-19) patients compared to healthy controls. Interestingly, in a study performed in peripheral blood mononuclear cells (PBMCs), Tian and colleagues reported a significant increase in *HIF1A* mRNA in COVID-19 patients compared to healthy controls. This difference in expression was also evident within the COVID-19 group, with elderly individuals presenting higher *HIF1A* mRNA expression [75]. It seems that in both ARDS and COVID-19, hypoxia is a primary driver of HIF stabilisation, which might amplify the inflammatory response.

Another important aspect in studies involving humans is the investigation of potential single-nucleotide polymorphisms (SNPs) with clinical significance. Yilmaz and colleagues focused on assessing *HIF1A* polymorphisms in paediatric ARDS patients. Their study demonstrated that the presence of the rs11549467 (G1790A) SNP was closely associated with the extent of the lung infiltration area in these patients [76]. Of note, the presence of the G1790A SNP has been linked with increased susceptibility to chronic obstructive pulmonary disease (COPD) and higher risks for lung cancer development, thus pinpointing its implication in pathological conditions [77,78]. Furthermore, in a recent yet-to-be-published preprint, Sipahioglu and colleagues investigated the clinical importance of the rs11549465 *HIF1A* SNP in patients who developed ARDS following COVID-19 infection. The investigators demonstrated that carriers of either the heterozygous CT or the homozygous TT genotype exhibited decreased 30-day mortality compared to the patients carrying the wild-type CC genotype. The clinical relevance of the rs480902 and rs516651 SNPs of the *PHD2* gene were evaluated, deriving no statistically significant results [79]. Both of the aforementioned *PHD2* gene polymorphisms had been previously examined by Dötsch and colleagues in a cohort of Caucasian ARDS patients. Specifically, it was shown that carriers of the homozygous TT genotype of the rs516651 *PHD2* SNP presented a 3.3-times greater risk for mortality [80]. Interestingly, a previous study in a cohort of Caucasian septic patients failed to reveal a correlation between the rs516651 *PHD2* SNP and 30-day mortality, thus emphasising its utility in patients with ARDS [81].

Circular RNAs (circRNAs) have also been linked to HIF signalling pathways in ARDS. A recent work published in 2022 demonstrated the connection between the HIF-1 signalling pathway and circRNAs with diagnostic potential. Specifically, Sun and colleagues examined the potential of circRNAs in the bronchoalveolar lung fluid (BALF) exosomes and the plasma of patients with ARDS. Among the plethora of characterised circRNAs, expression of circRNA002809 and circRNA103034 in both BALF exosomes and plasma exhibited a significant diagnostic ability in the identification of ARDS. Interestingly, both circRNAs were closely related to genes of the HIF-1 signalling pathway [82].

Table 2 presents a summary of HIF isoform expression in clinical studies involving critically ill patients with ARDS/COVID-19.

## 4. Targeting HIFs as Novel Therapeutic Strategies

ARDS is a complex and clinically heterogeneous syndrome, which partially explains the difficulty in effectively developing beneficial treatments [83]. To optimise the care of critically ill patients, there is a constant need for innovative therapies and advanced treatment strategies. HIF-1α has emerged as a potential therapeutic target in various human diseases [84], including sepsis, a heterogenous syndrome and a common cause of ARDS. In a recent review, Ruan and colleagues extensively summarised the potential therapeutic value of HIF-1α as a targeted therapy in the setting of sepsis [85].

To date, a plethora of drugs have presented promising data regarding the treatment of lung damage by regulating the expression of HIFs, either by upregulating or downregulating their expression. Compounds that induce HIF expression can be categorised as direct HIF inducers or PHD inhibitors, while targeted therapies for HIF downregulation comprise HIF activity inhibitors [85,86]. Several drugs that target the HIF pathway have been repurposed and evaluated for their therapeutic potential in ARDS. Since extensive reviews describing in detail various therapeutic strategies for HIF modulation in order to treat ALI and possibly ARDS have been published [87,88], we chose to focus on the most recent studies.

Han and colleagues examined the potential therapeutic effects of Roxadustat (FG-4592) in in vitro and in vivo LPS-induced lung injury models [89]. Roxadustat is a transient small-molecule PHD inhibitor that is used for the treatment of chronic kidney disease (CKD)-associated anaemia. Based on their results, HIF-1α exhibited a protective effect against LPS-induced acute lung injury. Roxadustat induced HIF-1α expression in the lungs through the HIF-1α/haeme oxygenase-1 (HO-1) signalling pathway, attenuating LPS-induced lung injury and inflammation, highlighting the protective role of HIF stabilisation in mitigating ALI [89]. In the same manner, FG-4497, which is a PHD2 inhibitor that mimics hypoxia and activates HIF-2α signalling, has been found to prevent pulmonary oedema and improve mortality in a sepsis-induced ALI mouse model. Specifically, endothelial cell-specific deletion of *EPAS1* in mice enhanced lung vascular permeability and oedema formation, which worsened in LPS- or CLP-induced lung injury. Treatment with FG-4497 activated HIF-2α-mediated transcription in a hypoxia-independent manner, leading to enhanced adherens junction integrity and preventing the loss of endothelial barrier function [90].

On a clinical basis, the investigation of several HIF PHD inhibitors has been completed or is currently underway in various stages of clinical trials [91]. Two phase II clinical trials that aimed to examine the efficiency of the HIF PHD inhibitors, Vadadustat and Desidustat, to prevent the progression of ARDS in hospitalised COVID-19 patients have been completed and their results are awaited [92,93]. In a recent review, Poloznikov and colleagues discuss the potential therapeutic benefits of HIF PHD inhibitors in COVID-19 patients with moderate to severe disease. They highlight that while they show great potential as a treatment for COVID-19, their role is complex since HIF-PHDs target multiple substrates, not just HIF, and, therefore, their effects may differ from those of hypoxia-stabilised HIF. Further research is needed to fully understand the potential therapeutic role of HIF–PHD inhibitors in COVID-19 [94]. 

Rabeprazole, a proton pump inhibitor that is used in acid reflux and ulcers, has been recently proposed as a potential therapeutic strategy for the treatment of sepsis and ARDS. Evans and colleagues have identified Rabeprazole as an HIF-1α inducer in lung endothelial cells. To determine whether Rabeprazole could improve inflammatory lung injury, they performed dose–response experiments in intraperitoneally LPS-injected mice. In a dose-dependent manner, Rabeprazole treatment 48 h post-LPS ameliorated lung oedema by reducing lung vascular permeability and neutrophil sequestration, and accelerating the resolution of sepsis-induced lung inflammation. The authors suggested that Rabeprazole promotes vascular repair and the resolution of inflammatory lung injury through the endothelial HIF-1α/FoxM1 signalling pathway [95].

Another compound that stimulates HIF-1α upregulation is acetate. Acetate is a short-chain fatty acid (SCFA) metabolite synthesised by the gut microbiota. Evidence suggests acetate as a potential therapeutic agent to enhance the host defence against pulmonary viral and bacterial infections [96,97]; however, the mechanism through which acetate induces its bactericidal activity remains elusive. Recently, Machado and colleagues examined the effect of acetate on *S. pneumoniae*-stimulated macrophages and presented a novel mechanism through which acetate reinforces the bactericidal activity of alveolar macrophages. They demonstrated that acetate enhanced the glycolytic activity of macrophages, resulting in HIF-1α activation and increased *IL1B* gene transcription. Simultaneously, acetate, through the NLRP3 inflammasome, promoted IL-1β secretion, which, in an autocrine manner, increased the production of nitric oxide (NO), leading to *S. pneumoniae* killing [98].

Inositol is a small polyol compound that could potentially relieve lung damage in ARDS. A study explored the role and mechanism of myo-inositol in the development of acute lung injury in an in vitro model of LPS-exposed human pulmonary alveolar epithelial cells and an in vivo model of intranasally LPS-challenged mice. Based on their results, inositol could enhance autophagy activation in the in vitro model to prevent pulmonary fibrosis progression via inhibiting the cell epithelial–mesenchymal transition (EMT) process mediated by the HIF-1α-SLUG axis [99].

Emodin, or *Rheum officinale*, is a natural anthraquinone with anti-inflammatory, antioxidant, antitumour, and vasodilating activities [100]. Li and colleagues investigated the effect of Emodin treatment in an LPS-induced ALI rat model and in an in vitro model using a mouse macrophage cell line exposed to LPS. Based on their results, Emodin treatment reduced the levels of inflammatory cytokines and downregulated the expression of HIF-1α in lung tissue through inhibiting the mammalian target of rapamycin (mTOR)/HIF-1α/vascular endothelial growth factor (VEGF) signalling pathway [101].

Chen and colleagues examined the effect of Norisoboldine on lung pathological changes in an LPS-induced mouse ALI model. Norisoboldine is the primary isoquinoline alkaloid of *Radix Linderae* and has anti-inflammatory, immunoregulating, and other biological activities. Norisoboldine treatment attenuated HIF-1α expression and hindered the translocation of M2 pyruvate kinase (PKM2) from the cytoplasm to the nucleus. Moreover, Norisoboldine ameliorated inflammation, decreased M1 polarisation of macrophages, and increased M2-polarised macrophages through the PKM2/HIF-1α/PGC-1α signalling pathway [102].

It seems that HIF targeting is promising in the development of novel therapeutic approaches against lung injury and ARDS [103]. HIF signalling pathways play a critical role in lung injury progression and vascular repair, making them potential therapeutic targets for diseases like COVID-19 and ARDS. However, it is important to highlight the complex nature of HIF signalling and the challenges associated with developing effective HIF-targeted therapies. For example, HIF-targeted therapies that induce systemic HIF activation could lead to undesirable increases in angiogenesis and endothelial cell proliferation. Moreover, it is important to monitor off-target adverse effects in distant and unaffected organs. Therefore, it is crucial to carefully consider the specific HIF isoform, target cell type, and timing of intervention to optimise HIF-targeted therapies [104]. 

## 5. Unravelling the Implication of HIF in Acute Lung Inflammation

In inflammatory processes, such as acute lung inflammation/ARDS, vascular leakage, oedema, and reduced oxygen diffusion can lead to localised hypoxia and contribute to HIF stabilisation in the inflamed lung. While hypoxia is a critical mediator of HIF expression, non-hypoxic stimuli, such as cytokines, ROS, and immune signalling pathways can also induce HIF activity, contributing to the overall inflammatory response.

HIF signalling pathways have a protective role by promoting cell proliferation, vascular repair, and the resolution of inflammation. However, HIF stabilisation may also amplify the inflammatory response, contributing to tissue damage and impaired healing, and can contribute to increased vascular permeability and worsened oedema. Hence, HIF plays dual roles in inflammation by promoting protective responses (e.g., enhancing barrier function and angiogenesis) and driving pathological processes (e.g., fibrosis and excessive inflammation).

The relationship between hypoxia and inflammation is complex; both can influence each other, depending on the specific pathological situation. Hypoxia enhances inflammation by stabilising HIF and promoting cytokine production, while inflammation worsens hypoxia by disrupting blood flow and increasing oxygen consumption. In acute lung inflammation, oedema and capillary damage cause hypoxia, which in turn amplifies the inflammatory response via HIF activation, perpetuating lung damage. 

Most often, lung-injured patients are treated with supplemental oxygen, which leads to hyperoxia. Hyperoxia suppresses HIF-mediated responses primarily by enhancing the degradation of HIF-α subunits, which can impair adaptive and reparative processes in lung-injured patients. However, the generation of ROS and other secondary effects complicate the overall impact. So, while supplemental oxygen is essential for treating hypoxaemia, prolonged hyperoxia can disrupt the complex balance of HIF-mediated protective and reparative mechanisms.

Gaining a deeper understanding of these delicate balances could provide valuable insights into the complex mechanisms driving acute lung inflammation, thus enabling more effective management strategies.

## 6. Conclusions

Progressive damage to the lung vasculature and endothelium during ARDS disrupts gas exchange, creating a hypoxic microenvironment that activates the HIF signalling pathway. In hypoxia, a key characteristic of ARDS, HIF signalling plays a dual role in both adaptation to and exacerbation of the condition. Although HIF targets include proinflammatory factors that can further propagate inflammation, stabilising HIF-1α is essential for managing pulmonary inflammation and oedema and promoting vascular repair. Given the divergent effects of HIF signalling, understanding its temporal dynamics in ARDS is crucial for developing therapies that target HIF pathways depending on the stage and severity of ARDS.

## Figures and Tables

**Table 1:** HIF-1 isoform expression in experimental models of acute lung injury.

HIF Isoform	Experimental Model	HIF Expression	References
HIF-1α	LPS mice	Increased mRNA and protein expression in lung tissue	[47]
	LPS mice	Increased protein expression in lung tissue	[48,49,50]
	LPS pigs	No changes in serum	[51]
	LPS A549 cells	Increased protein expression	[52]
	LPS isolated human neutrophils	Increased mRNA and protein expression	[53]
	CLP mice	Increased mRNA and protein expression in lung tissue	[54]
	CLP mice	Increased protein expression in lung tissue	[52,55]
	HCl aspiration mice	Increased mRNA expression in lung lysates	[56]
	HCl aspiration mice	Increased protein expression in lung lysates	[57]
	Ischemia reperfusion mice	Increased protein expression in lung tissue	[58,60]
	Ischemia reperfusion rats	Increased protein expression in pulmonary tissue	[59]
	Mechanical ventilation preterm lambs	Decreased protein expression in lung samples	[61]
	Mechanical ventilation mice	Increased protein expression in lung tissue	[62]
	Cyclic mechanical stretching Calu-3 cells	Increased protein expression	[62]
HIF-2α	LPS pigs	Decreased protein expression in serum	[51]
	CLP mice	Decreased mRNA expression in lung tissue	[54]
	Mechanical ventilation preterm lambs	Decreased protein expression in lung samples	[61]

A549, type II alveolar epithelial cell line; Calu-3, human airway epithelial cells; CLP, caecal ligation and puncture; HCl, hydrochloride; HIF-1α, hypoxia-inducible factor 1 alpha; HIF-2α, hypoxia-inducible factor 2 alpha; LPS, lipopolysaccharide.

**Table 2:** HIF-1α expression in critically ill patients with ARDS/COVID-19.

HIF Isoform	Overview of the Study	Findings on HIF Expression	Reference
	To explore the relationship of HIF-1α with the prognosis of ARDS. HIF-1α levels were measured in the sera of 98 ARDS patients, of whom 50 patients survived, and in 30 healthy volunteers	Increased protein expression in ARDS patients vs healthy controls. Increased protein expression in non-survivors vs survivors	[73]
HIF-1α	To examine whether HIF-1 is an early indicator of COVID-19 severity. HIF-1 was measured in the sera of 17 critically ill, 33 severe, and 40 mild/moderate COVID-19-induced ARDS patients and in 90 healthy volunteers	COVID-19 patients had significantly higher levels of HIF-1 than healthy volunteers. A progressive increase in HIF-1 serum levels was found according to ARDS severity in COVID-19 patients	[74]
	Whole-transcriptome RNA sequencing analysis was performed to explore altered gene expression mediated by SARS-CoV-2 infection. Isolated PBMCs from 11 COVID-19 patients and 9 healthy individuals	Increased mRNA expression in COVID-19 patients	[75]

ARDS, acute respiratory distress syndrome; COVID-19, coronavirus disease; HIF-1α, hypoxia-inducible factor 1 alpha; PBMCs, peripheral blood mononuclear cells; SARS-CoV-2, severe acute respiratory syndrome coronavirus 2.

## Data Availability

No new data were generated from this work.
